# Less Effort, Better Results: How Does Music Act on Prefrontal Cortex in Older Adults during Verbal Encoding? An fNIRS Study

**DOI:** 10.3389/fnhum.2014.00301

**Published:** 2014-05-12

**Authors:** Laura Ferreri, Emmanuel Bigand, Stephane Perrey, Makii Muthalib, Patrick Bard, Aurélia Bugaiska

**Affiliations:** ^1^Laboratoire d’Etude de l’Apprentissage et du Développement (LEAD), CNRS UMR 5022, University of Burgundy, Dijon, France; ^2^Movement to Health (M2H), EuroMov, Montpellier-1 University, Montpellier, France

**Keywords:** music, episodic encoding, fNIRS, prefrontal cortex, older adults

## Abstract

Several neuroimaging studies of cognitive aging revealed deficits in episodic memory abilities as a result of prefrontal cortex (PFC) limitations. Improving episodic memory performance despite PFC deficits is thus a critical issue in aging research. Listening to music stimulates cognitive performance in several non-purely musical activities (e.g., language and memory). Thus, music could represent a rich and helpful source during verbal encoding and therefore help subsequent retrieval. Furthermore, such benefit could be reflected in less demand of PFC, which is known to be crucial for encoding processes. This study aimed to investigate whether music may improve episodic memory in older adults while decreasing the PFC activity. Sixteen healthy older adults (μ = 64.5 years) encoded lists of words presented with or without a musical background while their dorsolateral prefrontal cortex (DLPFC) activity was monitored using a eight-channel continuous-wave near-infrared spectroscopy (NIRS) system (Oxymon Mk III, Artinis, The Netherlands). Behavioral results indicated a better source-memory performance for words encoded with music compared to words encoded with silence (*p* < 0.05). Functional NIRS data revealed bilateral decrease of oxyhemoglobin values in the music encoding condition compared to the silence condition (*p* < 0.05), suggesting that music modulates the activity of the DLPFC during encoding in a less-demanding direction. Taken together, our results indicate that music can help older adults in memory performances by decreasing their PFC activity. These findings open new perspectives about music as tool for episodic memory rehabilitation on special populations with memory deficits due to frontal lobe damage such as Alzheimer’s patients.

## Introduction

Research in cognitive aging has reported that older adults often present declines in specific memory systems (Tulving, 1972). Working memory, episodic memory, and prospective memory have been shown to substantially decline in the course of normal aging, while procedural memory and some perceptual memory functions show few age-related changes (Mitchell, 1989; Luo and Craik, 2008).

Episodic memory can be defined as the type of awareness experienced when one thinks back to a specific moment in one’s personal past and consciously recollects some prior episode or as it was previously experienced. This special kind of awareness is identifiable in all healthy and human adults (Wheeler et al., 1997). However, several studies have shown that healthy aging is often characterized by reduced access to contextually specific episodic memory details, resulting in larger deficits in source-memory performance than in item-memory performance (Craik et al., 1990; Glisky et al., 1995; Spencer and Raz, 1995; Dennis et al., 2008). Thus, older adults have difficulty remembering specific information about the circumstances under which an event was encountered, and they are less likely to remember the contextual features of events correctly (Johnson et al., 1993; Dodson et al., 2007), which can be considered to be a deficit in the ability to encode the spatio-temporal context of an event (Parkin and Walter, 1992; Bugaiska et al., 2007).

Several authors have observed that older adults produce lower scores than young adults on episodic memory tasks (mostly source-memory and recollection tasks) as a consequence of reduced executive function (Parkin and Walter, 1992; Bugaiska et al., 2007; see also Raz, 2000 and West, 1996). In particular, it has been claimed that impaired executive function reduces older adults’ ability to initiate the memory encoding of target information appropriately for a durable explicit representation (Bunce, 2003; Bugaiska et al., 2007). Furthermore, it has been found that low memory performance in older adults is related to both an associative deficit and a lower level of strategic functioning (Naveh-Benjamin, 2000; Shing et al., 2008). Neuroimaging and behavioral studies investigating cognitive aging have revealed that these deficits in episodic memory are related to prefrontal cortex (PFC) limitations due to reductions in hemispheric specialization of cognitive functions in the frontal lobes (e.g., Souchay et al., 2000; Craik and Grady, 2002; Luo and Craik, 2008). This effect has been conceptualized in terms of a model called the hemispheric asymmetry reduction in older adults (HAROLD) (Cabeza, 2002), and may be due to dedifferentiation of function, deficits in function, or functional reorganization and compensation in frontal lobe regions (Rajah and D’Esposito, 2005). In line with the idea of an age-related memory encoding deficit, several neuroimaging findings confirm episodic memory impairments linked to reduced recruitment of mediotemporal and PFC regions, mainly during the encoding phase of memory tasks (Daselaar et al., 2004; Dennis et al., 2008), supporting the hypothesis that older adults fail to encode target items thoroughly (Craik and Lockhart, 1972; Burke and Light, 1981; Isingrini et al., 1995).

Improving episodic memory encoding in spite of PFC deficits is therefore a critical issue in aging research. It is well-known that enriching the encoding context of an event, for example, through enacted encoding or with emotional valence stimuli, can enhance memory performance at retrieval (Hamann, 2001; Lövdén et al., 2002). Music is a complex auditory stimulus, which evolves over time and has a strong emotional impact (Blood and Zatorre, 2001) and thus engages the whole brain through different cognitive activities and neural substrates (Altenmüller, 2003). Consequently, music is likely to enrich the encoding of memory items and can thus be used to improve memory performance.

Although the role of background music in learning and memory tasks is still an open and debated question in the literature (Schellenberg, 2003; Schellenberg, 2005; De Groot, 2006; Peterson and Thaut, 2007; Jäncke and Sandmann, 2010), the role that music plays in terms of emotions (Jäncke, 2008), reward (e.g., Salimpoor et al., 2013), and positive arousal (e.g., Judde and Rickard, 2010) has brought several authors to believe that music can be used to improve memory encoding in a variety of situations. In particular, research in music and neuroscience has shown how music could boost verbal memory performance not only in clinical populations (Brotons and Koger, 2000; Ho et al., 2003; Thaut et al., 2005; Thompson et al., 2005; Racette et al., 2006; Franklin et al., 2008; Särkämo et al., 2008; Simmons-Stern et al., 2010), but also in healthy young and older adults (Balch et al., 1992; Wallace, 1994; Balch and Lewis, 1996; Thompson et al., 2005; De Groot, 2006; Ferreri et al., 2013; Kang and Williamson, 2013). However, there has been little research investigating whether background music affects the memory performance of older adults.

In a previous study on healthy young subjects (Ferreri et al., 2013), we used functional near-infrared spectroscopy (fNIRS) to investigate the role of background music on memory performance and the dorsolateral prefrontal cortex (DLPFC), which has been shown to play a crucial role in organizational, associative (Murray and Ranganath, 2007; Ranganath, 2010), and semantic (Innocenti et al., 2010) memory encoding, and particularly for organizational processing, which helps build associations among items that are active in memory (Blumenfeld and Ranganath, 2007). fNIRS is a non-invasive optical neuroimaging technique that can be used to monitor cortical activation during cognitive tasks through the well-characterized neurovascular coupling mechanism related to neuronal activation, namely, an increase in oxygenated (O_2_Hb) and a decrease in deoxygenated (HHb) hemoglobin concentrations (Jobsis, 1977; Ferrari and Quaresima, 2012). Moreover, fNIRS makes it possible to conduct more ecological cognitive experimental setups (subject sitting in a chair in a quiet room), which are not feasible in more traditional fMRI protocols, and it is thus suitable for investigating cortical activation in special populations such as older adults and neurological patients (Ferreri et al., in press). Recent fNIRS studies on verbal memory and learning have shown that facilitatory cues such as strategies (Matsuda and Hiraki, 2004, 2006; Matsui et al., 2007), pharmacological stimulants (Ramasubbu et al., 2012), or background music (Ferreri et al., 2013) during verbal encoding could result in deactivation, rather than greater activation, of PFC regions, suggesting less involvement/greater efficiency of high-cognitive functions mediated by PFC regions (such as the DLPFC) known to be crucial during memory encoding processes. In our previous experiment (Ferreri et al., 2013), we monitored bilateral DLPFC activation during verbal encoding with fNIRS and showed, for the first time, that background music during the encoding of verbal material can modulate DLPFC activity (i.e., decreased responses compared to a silent background) and, at the same time, facilitate the retrieval of the encoded material. We believe that these results open up interesting perspectives about how music could act on the DLPFC of people with memory disorders for whom the DLPFC is hypo-activated, impaired, or damaged, such as older adults or Alzheimer’s patients. Indeed, because encoding information with music decreases activation of the DLPFC, and because age-related differences in episodic memory are mediated by the decline of executive functioning supported by the PFC (Parkin and Walter, 1992; Bugaiska et al., 2007; Clarys et al., 2009), one could assume that encoding with background music would also lead to an improvement of episodic memory in older adults and more generally in populations with impaired or damaged PFC. Therefore, in the present study we explored the role of background music in the memory performance and DLPFC activation of healthy older adults through an item/source-memory paradigm and using fNIRS to monitor DLPFC activation bilaterally during verbal encoding with or without music. We hypothesized that music would enhance the encoding of verbal material in older adults by enriching the context and supplying organizational, associative, and semantic processes. Considering previous fNIRS studies showing better behavioral performance and PFC deactivation in response to facilitatory cues (Matsuda and Hiraki, 2004, 2006; Matsui et al., 2007; Ramasubbu et al., 2012), we therefore expected to find improved episodic memory performance at a behavioral level together with minimal demand on PFC activity during music encoding condition as compared to silence encoding condition. In particular, a classical activation pattern should be observed for the silence encoding condition, namely an increase of O_2_Hb and a decrease of HHb concentrations changes. On the other hand, we expected a DPLFC disengagement reflected in an inverse activation pattern, namely a decrease of O_2_Hb and an increase of HHb concentrations.

## Materials and Methods

### Participants

Sixteen healthy older adults, all right-handed, non-musicians, native French speakers (10 females, mean age 64.5 ± 2.5 years) volunteered to participate in the experiment. They scored above the 27-point cut-off on the mini-mental state examination (MMSE) to exclude dementia (Folstein et al., 1975). All the participants lived in their own homes, and reported themselves to be in good physical and mental health and to have normal or corrected-to-normal vision. All of them were recruited from the general “third age University” classes at the University of Burgundy and earn therefore a high-school degree. None were taking medication known to affect the central nervous system. Informed written consent was obtained from all participants prior to the experiment. The study conformed to the Helsinki Declaration, Convention of the Council of Europe on Human Rights and Biomedicine.

### Experimental procedure

The procedure was the same as the one used in our previous study (Ferreri et al., 2013). All the participants were seated at a computer in a quiet, dim room and carried out a memory encoding task while their DLPFC activation was monitored using fNIRS neuroimaging. They then performed a retrieval task. After the eight fNIRS channels had been adjusted on the forehead scalp overlaying the DLPFC and the in-ear headphones inserted, participants were informed that they would be presented with different lists of words with two auditory contexts: music or silence. They were explicitly instructed to memorize both lists and the context in which the words were encoded. The background music used in all blocks was an upbeat, acoustic jazz piece (“If you see my mother” by Sidney Bechet), chosen for its positive valence and medium arousal quality. Verbal stimuli consisted of 42 taxonomically unrelated concrete nouns selected from the French “Lexique” database (New et al., 2004, http://www.lexique.org), randomly divided into six lists (7 words per list, 21 words for each encoding condition), equated for word length and occurrence frequency.

**Figure 1:**
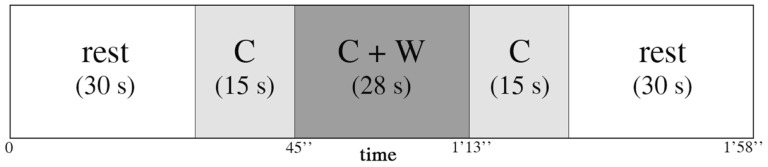
**Representation of one encoding block between two 30 s rest blocks**. Each block consisted of 15 s of context (C) alone, either music or silence in the earphones, then 28 s of context and words (C + W) encoding (seven words for each block, 4 s per word), and then again 15 s of context (C) alone.

The encoding phase consisted of three blocks of “music encoding” and three blocks of “silence encoding,” intermixed with 30-s rest periods. In each block, seven words were displayed successively with either a music or a silence background. The audio stimulation started 15 s before the first word was displayed, continued during the sequential display of words, and ended 15 s after the last word. Words in each block were presented at a rate of 4 s per word (28 s for the sequential presentation of seven words). Each block therefore lasted 58 s (15 s context, 28 s words, 15 s context) and was followed by a 30 s rest (silent) between each block (Figure 1). The order of music/silence blocks was counterbalanced, as were the order of the word lists and the order of words in the lists. During the rest periods, participants were instructed to try to relax and not think about the task; by contrast, during the context-only phases of the blocks (i.e., silence or music), they were instructed to concentrate on a fixation cross on the screen and to focus on the task. The entire encoding phase, together with fNIRS recording, took about 10 min.

Prior to the retrieval phase, participants performed two 5-min interference tasks: an “X–O” letter-comparison task (Salthouse et al., 1997) and a “plus–minus” task (Jersild, 1927; Spector and Bierdeman, 1976). They were then behaviorally tested for item and source-memory (Glisky et al., 1995). The retrieval test included the previously presented 42 words, together with 42 new words (lures) matched for word length and occurrence frequency. In a yes/no recognition task, participants were asked to say whether they had already seen each word before (yes/no button on the keyboard; item-memory task). If so, they were asked to indicate in which context they had seen it (music/silence/I do not know; source-memory task). In this way, we tested subjects’ capacity to remember specific episodes. The presentation of task instructions and stimuli, as well as the recording of behavioral responses, were controlled by E-Prime software (Psychology Software Tools, Inc.) running on a laptop with a 15′′ monitor.

### fNIRS measurements

An eight-channel fNIRS system (Oxymon Mk III, Artinis Medical Systems B.V., The Netherlands) was used to measure the concentration changes of O_2_Hb and HHb (expressed in micromolar) using an age-dependent constant differential path-length factor given by 4.99 + 0.0067 × (age 0.814) (Duncan et al., 1996). Data were acquired at a sampling frequency of 10 Hz. The eight fNIRS optodes (four emitters and four detectors) were placed symmetrically over the dorsal part of the PFC (Brodmann areas 46 and 9, EEG electrodes AF7/8, F5/6, F3/4 and AF3/4 of the international 10/10 system) (Okamoto et al., 2004; Jurcak et al., 2007), and the distance between each emitter and detector was fixed at 3.5 cm (Figure 3B).

To optimize signal-to-noise ratio during the fNIRS recording, the eight optodes were masked from ambient light by a black plastic cap that was kept in contact with the scalp with elastic straps, and all cables were suspended from the ceiling to minimize movement artifacts (Cui et al., 2011). During data collection, O_2_Hb and HHb concentration changes were displayed in real time, and signal quality and absence of movement artifacts were verified.

## Data Analysis

### Behavioral data

Each subject’s item- and source-memory accuracy (hit) rates (number of hits for each condition during yes/no recognition) and false alarms were calculated for both the silence and music conditions. Source-memory was determined as the proportion of correct source judgments among item-memory hits.

### fNIRS data

For each of the eight fNIRS measurement points, the O_2_Hb and HHb signals were first low-pass filtered to eliminate task-irrelevant systemic physiological oscillations (fifth order digital Butterworth filter with cut-off frequency 0.1 Hz).

In order to determine the amount of activation during the encoding phase for the two conditions, data for each of the six experimental blocks were baseline-corrected using the mean of the O_2_Hb and HHb signals during the last 5 s of “rest” block preceding each encoding block. We then sample-to-sample averaged (i.e., 10 samples/s) the baseline-corrected signals over the three blocks of each condition, yielding one average music and silence O_2_Hb and HHb signal per participant. In order to determine the level of DLPFC activation during the encoding phase for the two verbal encoding context conditions, we computed the maximum O_2_Hb and the minimum HHb values over the 28 s stimulus window (i.e., from *t* = 15 to 42 s), which indicated the maximum delta-to-baseline signal reached during the encoding phase.

Furthermore, in order to ascertain the DLPFC activation during the entire block of music/silence encoding conditions, we ran a complete time–series analysis in which we averaged O_2_Hb and HHb concentrations over 5 s windows (so one average point for each 5 s) all over the block of the encoding, getting 13 successive measures of concentrations (from the first seconds of fixation point preceding the words to the end of the block, with the last 5 s of rest phase taken as baseline value). Considering O_2_Hb increases and small HHb decreases as patterns of cortical activations (Jobsis, 1977), DLPFC activations were assessed by separately analyzing the baseline-corrected O_2_Hb and HHb concentration changes, and also the total hemoglobin concentrations (THb = O_2_Hb + HHb), for both the music and silence average block of each participant.

### Statistical analysis

For behavioral results, paired *t*-tests (one for each condition) were used to compare the item- and source-memory scores of the silence and music conditions. As well, paired *t*-tests were also used to test significant difference in false alarm rates for item and source-memory tasks, namely how many times they attributed a lure item (to have been) encoded with music or silence (music/silence item false alarms) and how many times subjects reported previously presented items (to have been) encoded in the wrong context (music/silence source false alarm). Furthermore, in order to account for bias with the source judgments, subjective source judgments were prorated according to prior item recognition. In this analysis, we took all the times where participants have said “music” (or “silence”) for the source, and looked at the number of correct items for this set. Paired *t*-tests were applied to estimate significant means difference for these values. One-sample *t*-tests were used to ascertain that all the scores were significantly above chance level.

For fNIRS results, the O_2_Hb, HHb, and THb concentrations were analyzed using a repeated-measures ANOVA with 2 (music/silence) × 2 (left/right hemisphere) × 4 (optodes) within subject factors, on which *post hoc* Bonferroni-corrected (confidence interval percentage = 99.3%) paired *t*-test comparisons determined which measurement points showed a significant difference between the two experimental conditions. Cohen’s *d* and Eta-squared statistics were used to calculate the effect size of paired *t*-tests and repeated-measures ANOVA, respectively. Concerning the time–series analysis, we ran a 2 (conditions) × 2 (left/right hemisphere) × 4 (optodes) × 13 (time points, namely successive measures of concentrations) repeated-measure ANOVA for both O_2_Hb and HHb values. Since SPSS software reports a partial Eta-squared value, which have been demonstrated to overestimate effect sizes (see Levine and Hullett, 2002), we calculated Eta-squared by dividing Type III Sum of squares of each condition and interaction by the corrected total (i.e., the sum of all Type III Sum of squares and error values computed in the statistics). The significance level was set at *p* < 0.05.

## Results

### Behavioral results

**Figure 2:**
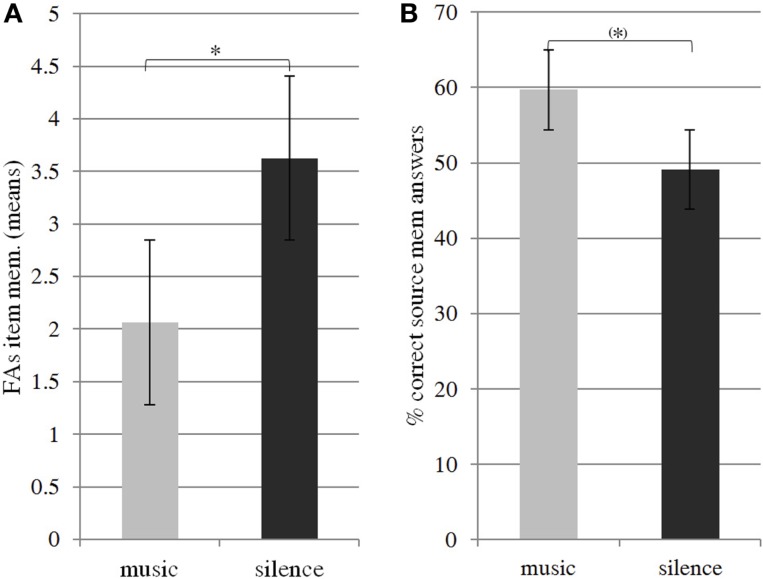
**Item-memory false alarm scores (A) and source memory correct answer scores (B)**. Paired *t*-tests showed fewer errors in item-memory performance and overall better source memory performance in the music condition than in the silence condition. One-sample *t*-tests revealed that music source memory scores were significantly above chance level, while silence source scores were not. * and (*) indicate, respectively, significant (*p* < 0.05) and marginally significant (0.05 < *p* < 0.07) difference in means.

Item-memory analysis revealed that subjects were significantly above chance level in yes–no recognition of items encoded with music and silence (one-sample *t*-test, *p* < 0.001). Whereas hits appeared constant across conditions for item-memory performance (*t* = −0.674, *p* = 0.302), there was a significant difference in false alarm rates (*t* = 2.498, *p* = 0.02, Cohen’s *d* effect size = −0.44), suggesting that background music during encoding led to fewer errors in the recognition phase (Figure 2A). The one-sample *t*-tests on source-memory scores revealed that while performance in the music condition was significantly above chance level (*t* = 3.537, *p* = 0.003), source-memory performance in the silence condition was below chance level (*t* = 0.418, *p* = 0.682), suggesting that participants had difficulty remembering words in the silence condition, and that they remembered words better in the music condition. Results obtained by considering subjective source judgments according to prior item recognition showed that item recognition was better (i.e., proportion of hits from all responses) if subjects during the retrieval phase judged the item as being presented with music rather than silence context during the encoding phase (*t* = 32.581, *p* = 0.021). Furthermore, a paired *t*-test revealed that the participants tended to retrieve the musical context better than the silence context (*t* = 31.57, *p* = 0.068). Taken together, behavioral results show that a musical background source not only plays an interfering role, but rather helps subjects during verbal encoding thus improving subsequent retrieval performances (Figure 2B).

### fNIRS results

**Figure 3:**
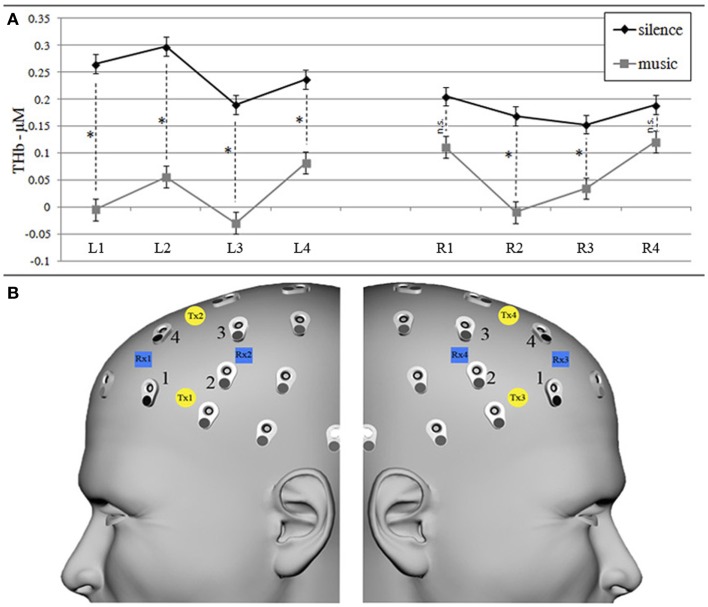
**Graphs of THb (A) concentration values (in micromoles) in silence and music conditions for each left and right fNIRS measurement channel (B)**. **(A)** Mean (±SEM) of THb (O_2_Hb + HHb) concentration values for silence (black line) and music (gray lines) conditions. * Shows significant (*p* < 0.05) within-channel differences obtained by *post hoc* Bonferroni-corrected paired *t*-test comparisons. **(B)** fNIRS transmitters (left Tx1–Tx2, right Tx3–Tx4, yellow circles) and receivers (left Rx1–Rx2, right Rx3–Rx4, blue squares) were placed on the left and right forehead scalp region, corresponding to AF7/8, F5/6, F3/4, and AF3/4 EEG channels (international 10/10 system), respectively, renamed left/right channels 1, 2, 3, and 4.

The repeated-measures ANOVA on bilateral DLPFC O_2_Hb values revealed a statistically significant main effect of condition [*F*(1, 15) = 8.390, *p* = 0.011], with greater bilateral O_2_Hb increases in the silence than in the music encoding condition and a significant condition × laterality interaction [*F*(1, 15) = 4.282, *p* = 0.056], with music presenting higher O_2_Hb values on the right hemisphere (although always lower than silence values). The strength of these effects computed by Eta-squared effect size revealed strong effect for the condition (η^2^ = 0.25) and weak for the interaction (η^2^ = 0.01). Furthermore, considering also HHb values, the repeated-measures ANOVA on THb values (Figure 3A) confirmed a main effect of condition [*F*(1, 15) = 14.329, *p* = 0.009, strong Eta-squared effect size = 0.22] in almost all channels, as shown by post hoc Bonferroni-corrected paired *t*-tests (Figure 3A). In line with several other fNIRS studies (e.g., Matsui et al., 2007; Okamoto et al., 2011), only HHb values did not show a significant effect.

**Figure 4:**
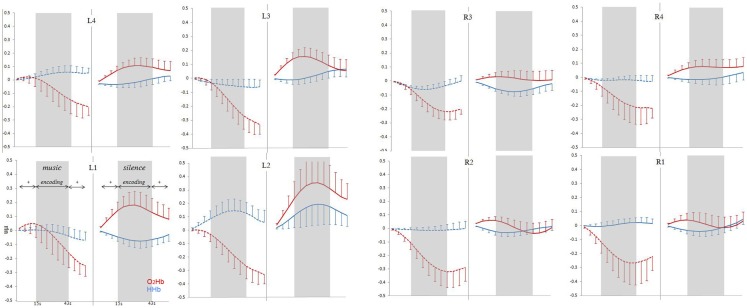
**Grand-average (±SEM) time-course of prefrontal cortex O_2_Hb (red lines) and HHb (blue lines) concentration changes (vertical axis) over the left and right hemisphere during memory encoding (horizontal axis: time) for the silence (right side, solid lines) and music (left side, dashed lines) conditions**.

Time–series analysis results are reported in Figure 4, which shows grand-average time-course of PFC O_2_Hb and HHb concentration changes at each of the eight fNIRS channels in the music and silence encoding conditions. The repeated-measure ANOVA on the O_2_Hb and HHb time-course series confirmed a main effect of condition [*F*(1,15) = 7.893, *p* = 0.013], corresponding to significantly greater O_2_Hb increases bilaterally in silence than music condition, and a marginally significant condition × laterality interaction [*F*(1,15) = 3.47, *p* = 0.082]. Interestingly, while the increases in O_2_Hb are visible bilaterally during the silence condition (and especially in left-hemisphere), time-course analysis revealed that the music condition was associated with a strong bilateral decrease of O_2_Hb lasting all over the block of the encoding (and affecting also the 15-s post-words fixation point in some channels).

## Discussion

Based on previous results with young adults (Ferreri et al., 2013), the present study aimed to investigate the neural mechanisms involved in memory–music processes (and the role of background music in particular), using fNIRS to monitor cortical response during the encoding phase among older adults, who usually show impairment in PFC activity and episodic memory tasks.

Our fNIRS results showed that the bilateral DLPFC was activated (O_2_Hb increase and HHb decrease) during the verbal encoding phase in the silence condition. Although recent studies suggest that caution should be exercised when applying fNIRS to infer PFC activation because of the task-evoked changes occurring in forehead skin perfusion (Kohno et al., 2007; Gagnon et al., 2011, 2012; Takahashi et al., 2011; Kirilina et al., 2012), these results confirm both the involvement of DLPFC during episodic memory encoding (Blumenfeld and Ranganath, 2007; Murray and Ranganath, 2007; Innocenti et al., 2010), and the suitability of fNIRS to investigate long-term memory processes (Kubota et al., 2006; Matsui et al., 2007; Okamoto et al., 2011; see also Cutini et al., 2012 for a review). The fNIRS results also showed a weak but significant interaction between memory encoding condition and hemispheric laterality, with greater left and right DLPFC activity (represented by O_2_Hb increases) in the silence and music conditions, respectively. This condition by laterality interaction can be explained by the presence of music during the verbal encoding phase, which could have shifted the classic left-verbal lateralization to the right hemisphere, in support of evidence that left-lateralization is determined by the nature of the material (verbal or non-verbal) being encoded (e.g., Kelley et al., 1998). However, compared to our previous fNIRS results with young subjects (Ferreri et al., 2013) which found marked left-hemisphere lateralization during both the music and silence encoding conditions (discussed in relation to the HERA model, Tulving et al., 1994; Nyberg et al., 1996), we observed a reduction in hemispheric asymmetry in the present study involving older adults. Furthermore, the weak significance revealed by O_2_Hb maximum values was not confirmed by time-course analysis, which revealed a marginal statistically significance, suggesting an absence of lateralization. This can be interpreted in relation to the HAROLD model (Cabeza, 2002), which predicts that DLPFC activity during cognitive performance tends to be less lateralized in older than in younger adults.

One of the main findings of the present study was that the eight measurement points surrounding the DLPFC made less demand on the DLPFC in the music than in the silence condition, resulting in decreased activity during the music verbal encoding phase (represented by an O_2_Hb decrease, see Figure 3). In view of previous fNIRS studies which showed decreased PFC activity during cognitive tasks (Matsuda and Hiraki, 2004, 2006), and specifically those related to verbal learning in which subjects were helped to memorize words by a given strategy (Matsui et al., 2007) or by a pharmacological stimulant (Ramasubbu et al., 2012), we previously discussed PFC disengagement during memory encoding with background music as evidence that music plays a facilitating, less-demanding role for the PFC during word encoding (Ferreri et al., 2013). In particular, we focused our argument on the fact that the PFC, specifically the DLPFC, is known to be recruited during cognitive tasks demanding organizational (Blumenfeld and Ranganath, 2007) and relational inter-item processing during encoding (Murray and Ranganath, 2007). Therefore, one possible interpretation of the DLPFC deactivation is that music helped older adults to generate inter-item and item-source relationships, without demanding high-cognitive PFC processes, which usually intervene when highly structured items (e.g., those which can be organized into chunks) are presented (see for example, Bor et al., 2003).

In other words, the presence of a musical background could affect memory by modulating the neurocognitive state, which facilitates the encoding, thus increasing subjects’ capacity to create associative bindings. Music could therefore modulate in a state-dependent manner the encoding mode, modifying the need of extra organizational and strategic encoding usually attributed to DPLFC, and facilitating the creation of richer associative links crucial for subsequent retrieval.

The behavioral results show that background music during the word encoding phase can assist memorization in older adults by reducing false alarm rates during recognition, confirming that item-memory performance can be improved by providing background music (Ferreri et al., 2013). Analysis of source-memory performance revealed that older adults could give details about the encoding context in the music condition more than in the silence condition, in which they were not able to retrieve the encoding context associated with the general information. These findings are in line with previous studies showing age-related impairments in remembering specific information about the circumstances under which a memory event was encountered (Johnson et al., 1993; Dodson et al., 2007). However, this difficulty was not encountered for the recollection of the musical context, suggesting that music could be a “good tool” to boost memory in older adults. Taken together, our results suggest that music can improve older adults’ episodic memory performance and, at the same time, demand less DLPFC activation.

It is therefore crucial to discuss these findings in the framework of aging research, and their possible implications for research in music cognition. In particular, these results can be seen more broadly as part of the ongoing debate about whether music can boost non-musical abilities, and more specifically verbal memory. Several studies have shown that not only musical training (Chan et al., 1998; Ho et al., 2003; Franklin et al., 2008), but also simple exposure to background music or sung stimuli leads to short- and long-term verbal memory benefits in healthy young and older adults (Balch et al., 1992; Wallace, 1994; Balch and Lewis, 1996; Thompson et al., 2005) and clinical populations such as stroke patients (Särkämo et al., 2008), people with multiple sclerosis (Thaut et al., 2005), aphasics (Racette et al., 2006), and Alzheimer’s patients (Thompson et al., 2005; Simmons-Stern et al., 2010). At the same time, it has also been claimed that music diverts participants’ attention from the items to remember by generating a dual-task situation and thus causing a perturbing effect on the memorization of verbal material (Salame and Baddeley, 1989; Racette and Peretz, 2007; Jäncke and Sandmann, 2010; Moussard et al., 2012; Jäncke et al., 2014). Thus, as recently pointed out by Kang and Williamson (2013), a delicate balance exists between music that facilitates recall from memory and music, which acts as a drain on limited memory resources. Our results seem to support the idea that music can benefit verbal memory in aging rather than generate a dual-task situation, in which it is well-known that older adults are usually penalized (see for example, Verhaeghen et al., 2003).

It is well-known that normal aging presents episodic memory deficits (e.g., Craik et al., 1990; Light, 1991; Craik and Grady, 2002). These impairments have been shown to be related to PFC dysfunction or reduced activity (Souchay et al., 2000; Cabeza, 2002; Craik and Grady, 2002; Luo and Craik, 2008), especially during the encoding processes (Daselaar et al., 2004; Dennis et al., 2008). In particular, older adults’ difficulty in episodic memory encoding has been shown to be related to an associative deficit, namely the difficulty in creating (and retrieving) cohesive episodes from unrelated attributes-units (Naveh-Benjamin, 2000), and also to a lower level of strategic functioning (Shing et al., 2008). The low episodic memory performance in our results could be related to associative deficits, which did not allow subjects to create appropriate strategies when encoding under the silence condition, obstructing the development of a durable memory trace. Interestingly, it has also been shown how this associative deficit in older participants can be circumvented by using appropriate associative strategies (e.g., creating a sentence linking pairs of words during encoding), which resulted in a significant improvement in episodic memory performance (Naveh-Benjamin et al., 2007). One possible explanation could therefore be that music helps older subjects to create strategies of associations during the encoding phase (between items and between the item and the source itself), which are not easily created under the silence condition, and in this way improves subsequent episodic memory performance. In line with this idea, it has been shown that certain situations can provide additional elaborative encoding, but it is only when using effortful processing, such as associative and integrative processing (Chalfonte and Johnson, 1996; Naveh-Benjamin, 2000), that older adults might find difficulty (Luo et al., 2007). A study by Luo et al. (2007) tested this hypothesis by devising a condition in which visually presented words were paired with an appropriate sound. Results showed that older adults benefited very little from associating sound to words, because of the additional demand this places on integrative processing. Thus, one possible reason for the enhancement of memory in a music condition is that it does not require effortful processing. This in turn would provide a mechanism to explain why less DLPFC activity was required for verbal encoding with background music, which, unlike the silence condition, does not require DLPFC involvement for the high-cognitive processing of verbal encoding, suggesting more automatic inter-item/item-source binding when there is background music. This explanation is supported by previous EEG findings that a few seconds of music can influence the semantic and conceptual processing of verbal material by priming the meaning of a word (Koelsch et al., 2004; Daltrozzo and Schön, 2009a,b). It is therefore possible that this semantic priming mechanism could also be reflected in easier associations and bindings between items when there is background music, thus requiring less demand on the DLPFC.

However, it is important to mention that a consistent part of the literature on episodic memory has shown how an item is better encoded when supported by semantically related contextual stimulation (see e.g., Engelkamp and Zimmer, 1984; Light, 1991; Lövdén et al., 2002). In our case, we have chosen a “Jazz” music piece, which could be a rich and pleasant encoding context for the subject, but that is not semantically related with the encoded items. Considering this, one could claim that the observed results are related to an increase of attention rather than due to associative and binding strategies linked to the background music. It has been discussed that, because of its intrinsic arousal potential, music might represent a powerful exogenous means of memory modulation, especially for long-term memory processes (Judde and Rickard, 2010). By comparing a musical versus non-musical context, it is therefore possible that music just improves subjects’ attention thus increasing their performance. In this case, our findings would be line with previous behavioral studies, which attributed improved cognitive performance in the presence of a musical background to higher amounts of arousal and attention (Foster and Valentine, 2001; Thompson et al., 2005). In apparent conflict with this explanation there is the fact that, music being considered as an unrelated context, we should expect an attentional overload (with the attention divided between musical auditory stimulus and verbal encoding task) and observe music as an interference rather than an attention improving factor. This would be in line with the literature, which claims that music draws participants’ attention away from the relevant information to remember thus creating a dual-task situation (e.g., Salame and Baddeley, 1989; Racette and Peretz, 2007; Jäncke and Sandmann, 2010; Moussard et al., 2012; Jäncke et al., 2014). In other words, we should have observed worse memory retrieval performance for the “unrelated” music condition when compared to silence condition which, even if semantically unrelated to the stimuli, allows the subjects to freely focus on the task without overloading their attentional field. This would be especially true for older adults, known to have impairments in inhibiting irrelevant information and therefore in dividing their attention during encoding (e.g., Park et al., 1989; Parks, 2007). However, in line with previous studies showing that background music (even when semantically unrelated) can help non-musical cognitive abilities (e.g., Balch et al., 1992; Wallace, 1994; Balch and Lewis, 1996; Thompson et al., 2005; De Groot, 2006; Ferreri et al., 2013; Kang and Williamson, 2013), our behavioral results show that music was not interfering with the memory encoding task, but rather helped subjects when presented as an encoding context. Furthermore, if an attentional explanation would be true, we should have expected our functional neuroimaging results to show greater PFC involvement in the music condition than in the silence condition. Several studies have indeed shown how alertness or attentional states significantly increase PFC activation (Ehlis et al., 2008; Herrmann et al., 2008), and these findings do not match with the decreased PFC activation found in our fNIRS data for the music encoding.

Another important point is that the PFC deactivation we found is in apparent conflict with previous fMRI study which showed that PFC, specifically the DLPFC, is recruited during cognitive tasks demanding inter-item organizational and strategic encoding (Blumenfeld and Ranganath, 2007; Murray and Ranganath, 2007) and that its activity usually increases when highly structured items are presented (e.g., Bor et al., 2003). As discussed by Ramasubbu et al. (2012), such PFC deactivation is in line with previous PET studies (e.g., Volkow et al., 2002) and the discrepancies between fMRI and near-infrared spectroscopy (NIRS) studies could be in part due to differences in methodologies and wide variation in correlation between O_2_Hb–HHb and blood oxygen level-dependent (BOLD) responses (see e.g., Cui et al., 2011 for a combined fMRI–fNIRS study). However, it is reasonable to think that DLPFC deactivation goes with activation of other brain regions, and particularly subcortical regions. Starting from these considerations, although fNIRS spatial resolution limitations do not allow monitoring subcortical oxygenation changes, it is interesting to discuss another possible explanation for our findings, which relies on music-related emotional factors. It is well-known that a strong relationship exists between music and limbic-reward systems; music is indeed one of the most potent motivational rewards and several studies showed that pleasant music experiences are accompanied by activity in subcortical brain regions (e.g., ventral–tegmental area and nucleus accumbens) involved in motivation and reward processes (see e.g., Blood and Zatorre, 2001; Salimpoor et al., 2013). At the same time, it has also been demonstrated that mesolimbic reward circuit activations can precede memory formation during reward-motivated learning, suggesting the crucial role that the reward system plays in successful memory encoding processes (Adcock et al., 2006). It is therefore possible that the observed PFC disengagement is linked with implicit subcortical mesolimbic music-related activation, which most likely improved subjects’ items learning and therefore the subsequent memory performance.

Further research on both behavioral (e.g., specific organizational and associative strategies, musical semantic related and unrelated material, pleasant and unpleasant music) and functional (e.g., with multi-channel fNIRS systems during both encoding and retrieval phases) levels may confirm these explanations and shed new light on which cognitive processes are involved during episodic encoding with music. However, in our opinion, these findings contribute to the ongoing debate about which neural mechanisms underlie the therapeutic effects of music. In particular, we believe that our results open up interesting perspectives about the use of music as a rehabilitation tool for people with memory deficits due to frontal lobe dysfunctions, such as Alzheimer’s patients. The notion that memory for music can be preserved in patients with Alzheimer’s disease has been raised by a number of case studies (Baird and Samson, 2009), and several studies have shown that the verbal memory of Alzheimer’s patients can be improved by music (Thompson et al., 2005; Simmons-Stern et al., 2010). However, the processes underlying these improvements remain unclear. To investigate the effect of music on episodic performance in special populations such as Alzheimer’s patients while non-invasively monitoring their PFC brain oxygenation through fNIRS could therefore offer a great opportunity to clarify how music could act on long-term memory processes and to understand better how and why music works in rehabilitation.

In conclusion, in line with the view that context is crucial during verbal episodic memory encoding, our findings suggest that music can create a rich and helpful context during the encoding of verbal material, improving the subsequent episodic memory performance of older adults, and that this improvement goes alongside less involvement of the PFC. Taken together, these results are in line with the idea that music is a good tool for memory rehabilitation and open up new perspectives about the cortical mechanisms involved in the therapeutic effects of music.

## Conflict of Interest Statement

The authors declare that the research was conducted in the absence of any commercial or financial relationships that could be construed as a potential conflict of interest.
